# A Re-examination of the Effect of Masker Phase Curvature on Non-simultaneous Masking

**DOI:** 10.1007/s10162-017-0637-5

**Published:** 2017-08-23

**Authors:** Robert P. Carlyon, Sheila Flanagan, John M. Deeks

**Affiliations:** MRC Cognition & Brain Sciences Unit, University of Cambridge, 15 Chaucer Rd, Cambridge, CB1 3DA UK

**Keywords:** phase curvature, forward masking

## Abstract

Forward masking of a sinusoidal signal is determined not only by the masker’s power spectrum but also by its phase spectrum. Specifically, when the phase spectrum is such that the output of an auditory filter centred on the signal has a highly modulated (“peaked”) envelope, there is less masking than when that envelope is flat. This finding has been attributed to non-linearities, such as compression, reducing the average neural response to maskers that produce more peaked auditory filter outputs (Carlyon and Datta, J Acoust Soc Am 101:3636–3647, 1997). Here we evaluate an alternative explanation proposed by Wotcjzak and Oxenham (Wojtczak and Oxenham, J Assoc Res Otolaryngol 10:595–607, 2009). They reported a masker phase effect for 6-kHz signals when the masker components were at least an octave below the signal frequency. Wotcjzak and Oxenham argued that this effect was inconsistent with cochlear compression, and, because it did not occur at lower signal frequencies, was also inconsistent with more central compression. It was instead attributed to activation of the efferent system reducing the response to the subsequent probe. Here, experiment 1 replicated their main findings. Experiment 2 showed that the phase effect on off-frequency forward masking is similar at signal frequencies of 2 and 6 kHz, provided that one equates the number of components likely to interact within an auditory filter centred on the signal, thereby roughly equating the effect of masker phase on the peakiness of that filter output. Experiment 3 showed that for some subjects, masker phase also had a strong influence on off-frequency *backward* masking of the signal, and that the size of this effect correlated across subjects with that observed in forward masking. We conclude that the masker phase effect is mediated mainly by cochlear non-linearities, with a possible additional effect of more central compression. The data are not consistent with a role for the efferent system.

## **INTRODUCTION**

For many decades, models and accounts of masking were based on the power spectrum of the masker and signal, as processed by the amplitude characteristics of a bank of putative auditory filters, and on the signal-to-noise ratio at the outputs of those filters (Fletcher, 1940; Patterson, 1976; Glasberg and Moore, 1990). Although such models account for a wide range of data, more recent accounts have focussed on a number of important failures of the power spectrum model. These include the effects of masker uncertainty and other higher-level aspects of sound (“informational masking”), the effects of masker-to-signal onset delay in simultaneous masking (i.e. the “overshoot effect”), and the non-linear growth of masking observed when the signal has a frequency substantially higher than that of the masker (Zwicker, 1965; Neff and Callaghan, 1987; Jennings et al., 2011; Yasin et al., 2013). In contrast to the operational flavour of early models, contemporary accounts of auditory processing are often inspired by, and explicitly include, biological phenomena such as cochlear non-linearity, neural adaptation, and, sometimes, the operation of the efferent system (Patterson et al., 1995; Lopez-Poveda and Meddis, 2001; Meddis and O'Mard, 2006; Wojtczak and Oxenham, 2009; Jennings et al., 2011).

The present study is concerned with an important phenomenon that has inspired considerable experimentation aimed at unearthing its physiological basis (Smith et al., 1986; Kohlrausch and Sander, 1995). Smith et al. (1986) compared the simultaneous masking of a pure tone by two maskers that had identical power spectra and consisted of *N* consecutive components of a harmonic complex. The components of the two maskers were summed in so-called positive and negative Schroeder phase (Schroeder, 1970), corresponding to values of +1 and −1 for the parameter *C* in Eq. 1, where *N* is the total number of components and *θ*_n_ is the phase of the *n*th component:

1 $$ {\theta}_{\mathrm{n}}= C\pi n\left(n-1\right)/N $$

For sinusoidal signals longer than the period of the masker, the masker with the positive curvature (*C* = 1; “*S*+”) produced less masking than the one with the negative curvature (*C* = −1; “*S*−”). Similar findings were subsequently obtained by Kohlrausch and Sander (1995), who also found that thresholds for brief tones varied markedly throughout the period of the *S*+ but not the *S*− masker. They concluded that the *S*+ masker had a phase curvature that was opposite to that of the auditory filter centred on the signal frequency, which therefore must be negative. They proposed that this led to components having roughly equal phase at the output of the auditory filter, leading to a “peaked” response. This result has since been replicated and extended to other levels and probe frequencies (Carlyon and Datta, 1997b; Summers and Leek, 1998; Summers, 2000; Oxenham and Dau, 2001a, b, 2004). Subsequent studies have obtained a more fine-grained estimate of auditory filter phase curvature by measuring masking for a number of values of *C* in Eq. 1 (Lentz and Leek, 2001; Oxenham and Dau, 2001b; Oxenham and Ewert, 2005).

When the masker and signal are presented simultaneously, differences in masking produced by *S*+ and *S*− maskers could arise for several reasons. These include listening in the dips in the modulated neural response to the *S*+ masker and the operation of cochlear non-linearities such as the greater suppression of the probe tone by *S*− than by *S*+ maskers (Recio and Rhode, 2000; Oxenham and Dau, 2004). Carlyon and Datta (1997a) argued that forward masking would not be influenced by dip listening, and that a comparison of forward masking by *S*+ and *S*− complexes could provide an estimate of the average amount of excitation produced by each masker in the auditory filter centred on the signal and therefore of fast-acting compression in the auditory system. They reasoned that, when the auditory filter output was highly peaked, compression would reduce the amplitude of those peaks, and that this effect would be larger than for a stimulus where the auditory filter outputs had a flat envelope. Consistent with this prediction, forward-masked thresholds were substantially lower for the *S*+ masker than for the *S*− masker. The size of this difference was greatest at the highest masker level (69-dB SPL/component) tested and decreased at lower masker levels. Carlyon and Datta noted that this was consistent with a role for basilar-membrane compression, which is also reduced at low levels. However, they also noted that auditory filter bandwidths are narrowest at low levels, and that the peaked filter outputs require the interaction of many components and therefore a wide filter bandwidth. They additionally pointed out that their results could be influenced by fast-acting compression at any stage of auditory processing, provided that the compression was faster than the 10-ms period of their maskers and prior to the processing stage at which detection occurred. Subsequently, Gockel et al. (2003) reported greater forward masking by a random-phase than by a cosine-phase harmonic complex and interpreted their results in terms of the cosine-phase complex undergoing greater compression and, additionally, greater mutual suppression between the masker components. Because compression and suppression are manifestations of the same basilar membrane (BM) non-linearity, we will use the term “BM non-linearity” throughout most of the rest of this article and will discuss the relationship between the two phenomena in the “Discussion.”

More recently, Wojtczak and Oxenham (2009) proposed an alternative mechanism that could lead to effects of masker phase on forward-masked thresholds, without those phase effects necessarily influencing the amount of masker excitation at the signal frequency. They measured the effect of phase curvature for signal frequencies of 1, 2, and 6 kHz, both for on-frequency maskers, where the masker spectrum encompassed the signal frequency, and off-frequency maskers, where the frequencies of the masker components were all below the signal frequency. The results for the on-frequency maskers replicated and extended Carlyon and Datta’s (1997a) results. The off-frequency maskers also showed a strong effect of phase curvature but only for the 6-kHz signal frequency, even though the highest masker component was more than half an octave below that of the signal. This effect was reduced when the masker duration was reduced from 200 to 30 ms. Wojtczak and Oxenham (2009) argued that this off-frequency phase effect could not be explained by BM compression, because of evidence that the BM response at a given place is linear when driven by sufficiently lower-frequency components (e.g. Ruggero et al., 1997). They also dismissed an explanation in terms of fast-acting compression central to the BM, because this could not explain the fact that no phase effect was observed for off-frequency maskers at low signal frequencies. Instead, they attributed the effect of phase on off-frequency masking to the operation of the medial olivocochlear reflex (MOCR), whose activation was proposed to be dependent on the response of auditory nerve fibres tuned to the masker. They assumed that this activation was greater when that “internal response” had a flat envelope than when it had a peaky envelope, and that, for the 200-ms masker, this MOCR activation reduced the neural response to the signal. The fact that the phase of off-frequency maskers affected forward masking only at the 6-kHz signal frequency was explained by citing evidence for greater efferent activation at high frequencies (Kawase et al., 1993; Kawase and Liberman, 1993). Note that, according to this explanation, masker phase could affect the response to the signal without necessarily affecting the average amount of neural activity elicited by the masker in the auditory filter centred on the signal. Another potential mechanism, the middle-ear muscle (MEM) reflex, could in principle also allow masker phase to influence thresholds by virtue of its effect on masker excitation in a frequency region remote from that of the signal. A subsequent study (Wojtczak et al., 2015) combined behavioural measures with recordings of oto-acoustic emissions. The results were complex to interpret, and the authors concluded that there was no strong evidence either for an effect of the MOCR or of the MEM reflex.

The present study provides a further investigation of the effects of phase curvature on non-simultaneous masking and, in particular, investigates the potential role of the efferent system. It imposes some further constraints on the possible physiological mechanisms and reaches conclusions that differ from those of Wojtczak and Oxenham (2009). Experiment 1 replicated their main findings, including the effect of masker phase curvature and duration on the off-frequency masking of a 6-kHz signal in forward masking. This off-frequency effect was variable across listeners but statistically significant. Experiment 2 re-examined the conclusion that off-frequency effects only occur at high signal frequencies. By manipulating the fundamental frequency (F0) of off-frequency maskers, it showed that a substantial phase effect can be obtained at lower signal frequencies, provided that there are enough components in the off-frequency masker. Experiment 3 measured the effect of phase curvature of an off-frequency masker on the detection of a 6-kHz signal in *backward* masking. The phase effect was also variable across listeners but correlated with the effect of phase on forward masking in the same group of listeners. This cannot be due to the operation of the MOCR or of the MEM. We conclude that although the exact mechanism underlying the effect of masker phase on forward-masked thresholds are not known, the data are consistent with a combination of the BM non-linearity and, possibly, more central compression.

## **EXPERIMENT 1**

### Methods

Experiment 1 replicated key aspects of the study by Wojtczak and Oxenham (2009), using their highest masker level of 85 dB SPL at which the largest masker phase effects were observed. Two signal frequencies, 1 and 6 kHz, were tested. The signal had a duration of 10 ms, consisting of two 5-ms raised-cosine ramps, and it was presented immediately after the 200- or 30-ms maskers, which were also turned on and off with 5-ms raised-cosine ramps. Maskers had an F0 of 100 Hz and were either “on-frequency” or “off-frequency.” Their frequency content, which was the same as in Wojtczak and Oxenham (2009), is described in Table 1. Masker components were added in the phase described in Eq. 1 with *C* set to −1 and +1 for the 1-kHz signal and to −1 and 0 for the 6-kHz signal; we sometimes refer to these as the “flat” and “peaky” stimuli, respectively. These values were selected so as to produce the maximum phase effects based on the results of Wojtczak and Oxenham (2009), who obtained masked thresholds for nine values of *C* ranging from −1 to +1. The starting phase of the components was randomised from presentation to presentation by generating a stimulus that was one period (10 ms) longer than that presented and by selecting the start point at random over a 10-ms window. This randomization was also applied in all other experiments described here. Thresholds were measured using a three-interval three-alternative forced-choice task and a two-down one-up adaptive procedure (Levitt, 1971). Feedback as to correct answers was given after each trial, via a computer monitor. The step size in the adaptive procedure was 8 dB for the first two turnpoints, 4 dB for the next two turnpoints, and 2 dB for the remaining eight turnpoints of each procedure. Thresholds for each run were calculated from the mean of the last eight turnpoints. Each threshold reported here was obtained from the mean of at least four adaptive runs per subject. Five normal-hearing (see below for details) subjects took part, and their average data are presented. Subjects were seated in a sound-attenuating booth and made responses via computer mouse and monitor. Conditions were run in a counter-balanced order.

**TABLE 1:** Maskers used in experiment 1. The *left-hand column* shows the signal frequency in kHz. The *next four columns* show the number of masker components, the lowest and highest masker component frequency (Hz) and the masker frequency range for the on-frequency condition (Hz). The corresponding values for the off-frequency condition are shown in the *next four columns* followed by the two values of *C* (Eq. 1) tested at each frequency

Fs (kHz)	On-frequency				Off-frequency				*C*
	*N*	Low	High	Range	*N*	Low	High	Range	
1	13	400	1600	1200	6	100	600	500	−1, +1
6	25	4800	7200	2400	25	1600	4000	2400	−1, 0

All stimuli were generated digitally at a sampling frequency of 50 kHz. The maskers and probes were played out of separate channels of a CED 1401plus D/A converter (Cambridge Electronic Design, Cambridge) and attenuated by separate programmable attenuators (Tucker-Davis-Technologies (Alachua, Florida, USA), System 2). They were then summed using a custom-built headphone amplifier and sent to the left earpiece of a Sennheiser HD650 headphone. All stimuli were calibrated using a Kemar Type 45DA head assembly containing a G.R.A.S. Type 40 AG microphone, with microphone output measured with an HP3561A dynamic signal analyser.

All subjects were aged between 19 and 41 years. The left ear was used for all experiments. Prior to the main experiments, pure tone thresholds were measured for each subject (left ear), using a two-interval two-alternative forced-choice task and a two-down one-up adaptive procedure. A total of 16 turnpoints were measured, with the last 12 turnpoints averaged to represent threshold for the run. Between 1 and 4 runs were averaged to represent threshold for each tone frequency. Thresholds were determined for 0.5, 1.0, 2.0, 3.0, 4.0, 6.0, and 8.0-kHz pure tones. For these measures only, stimuli were 300 ms in duration inclusive of 10-ms raised-cosine ramps. Tones were presented using an Asus Xonar Essence STX soundcard using a sample rate of 44.1 kHz, via a custom-built headphone amplifier. The level of tones was controlled with a Tucker-Davis-Technologies PA4 programmable attenuator, with the level step set to be 5 dB for the first four turnpoints of each procedure and 1 dB for the last 12 turnpoints. Stimuli were presented via the left ear piece of Sennheiser HD650 headphones. For all subjects, average thresholds for each tone frequency were below 15 dB HL, apart from two subjects (S1 and S3) with thresholds of 17.3 and 21.0 dB HL at 8.0 kHz, respectively.

### Results

**FIG. 1:**
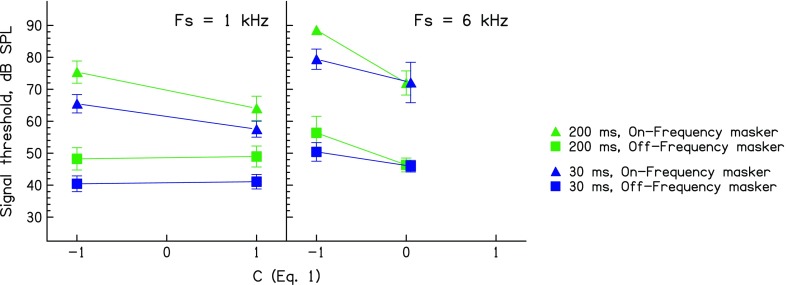
Forward-masked signal detection thresholds, as a function of masker phase curvature (“*C*”). Data for the 1- and 6-kHz signal frequencies are plotted in the *left-* and *right-hand panels*, respectively. Thresholds obtained with on-frequency maskers are shown by *triangles*; those for off-frequency maskers are shown by *squares*. *Green symbols* and *lines* represent data obtained with 200-ms maskers whereas those obtained with 30-ms maskers are shown in *blue*. *Error bars* show ±1 standard error of the group mean.

The results of experiment 1, averaged across listeners, are plotted in Figure 1. They replicate several key findings described previously (Carlyon and Datta, 1997a; Wojtczak and Oxenham, 2009). For the 1-kHz signal, the flat masker (*C* = −1) produces substantially more masking than the peaky masker (*C* = 1) in the on-frequency condition (triangles) but not in the off-frequency condition (squares). As expected, on-frequency maskers produced more masking overall than off-frequency maskers, and the 200-ms maskers were more effective than the 30-ms maskers. Furthermore, masker phase influenced thresholds only for the on-frequency masker. All of these effects were confirmed by a three-way (duration × phase × masker frequency range) repeated-measures ANOVA (phase *F*(1,4) = 70.8, *p* = 0.001; duration *F*(1,4) = 55.5, *p* < 0.01; phase × masker frequency range *F*(1,4) = 88.4, *p* = 0.001; masker frequency range. *F*(1,4) = 126.2, *p* < 0.001). No other main effects or interactions were significant.

For the 6-kHz signal, there was also more masking by the flat (*C* = −1) masker than by the peaky (*C* = 0) masker (*F*(1,4) = 25.6, *p* < 0.05), and on-frequency maskers were more effective than off-frequency maskers (*F*(1,4) = 67.0, *p* < 0.001). Also, as was the case for the 1-kHz signal, there was a significant phase × masker frequency range interaction, reflecting a larger effect of masker phase for on-frequency (triangles) than off-frequency (squares) maskers (*F*(1,4) = 14.2, *p* < 0.05). As was the case at 1 kHz, the effect of masker duration was significant (*F*(1,4) = 46.8. *p* < 0.05). However, unlike at 1 kHz, this duration effect interacted with masker phase, reflecting the fact that the effect of phase was greater for 200-ms than for 30-ms maskers (*F*(1,4) = 11.7, *p* < 0.05). One prediction of Wojtczak and Oxenham’s explanation is that this interaction would be more pronounced for off-frequency maskers, for which the effect of phase on thresholds is assumed to depend on the MOCR, which does not have time to act for the 30-ms masker. That is, the effect of masker phase on thresholds should depend more strongly on duration for off-frequency than for on-frequency maskers. We could not find statistical evidence for this prediction (frequency region × duration × phase interaction (*F*(1,4) = 1.5, *p* = 0.2)). The trend was numerically true in the study by Wojtczak and Oxenham (2009) but was not tested statistically in that study.

To evaluate differences in the pattern of results between the two signal frequencies, we performed additional repeated-measures ANOVAs with signal frequency as a factor. In this case, we entered masker phase as a single factor which we describe as “peaky vs. non-peaky,” even though these correspond to different values of *C* for the two signal frequencies. Our rationale for doing so is to compare the maximum possible effects of phase curvature at the two signal frequencies, and that the values of *C* that we used were chosen using Wojtczak and Oxenham’s (2009) data so as to maximise the phase effect at each frequency. This four-way ANOVA (signal frequency × flat vs. peaky masker × masker frequency region × duration) provided statistical evidence for one finding important for Wojtczak and Oxenham’s hypothesis, which is that the effect of duration on the masker phase effect was greater at the 6-kHz than at the 1-kHz signal frequency; there was a significant interaction between masker phase, masker duration, and signal frequency (*F*(1,4) = 9.4, *p* < 0.05). This ANOVA also confirmed the main effects of masker phase (*F*(1,4) = 40.7,*p* < 0.01), masker duration (*F*(1,4) = 220.3, *p* < 0.001), and masker frequency range (*F*(1,4) = 127.1, *p* < 0.001). In addition, the effect of masker phase was larger at the higher signal frequency, consistent with more components interacting within the broader auditory filter at 6 kHz than within the narrower 1-kHz filter (frequency × phase interaction *F*(1,4) = 11.2, *p* < 0.05). As was the case for the individual signal frequencies, masker phase had a larger effect for the on-frequency than for the off-frequency maskers (phase × masker frequency range: *F*(1,4) = 41.7, *p* < 0.01), and this effect depended somewhat on masker duration (phase × masker frequency range × duration: *F*(1,4) = 14.5, *p* < 0.05).

Perhaps the most crucial finding for Wojtczak and Oxenham’s hypothesis and the motivation for our experiment 2 is whether the effect of masker phase for off-frequency maskers was greater at 6 kHz than at 1 kHz. Inspection of Figure 1 suggests that masker phase did indeed have an effect on thresholds for the off-frequency masker at 6 kHz but not at 1 kHz. This was supported by an additional three-way ANOVA on the off-frequency data only, which showed a significant interaction between signal frequency and masker phase (*F*(1,4) = 10.4, *p* < 0.05).

## **EXPERIMENT 2. OFF-FREQUENCY FORWARD MASKING**

### Method and Rationale

Experiment 1 found that the effect of masker phase for off-frequency maskers was larger for a 6-kHz than for a 1-kHz signal frequency. This replicated the finding by Wojtczak and Oxenham (2009), who also found that the off-frequency phase effect at a signal frequency of 6 kHz was larger than at 1 kHz. They argued that compression central to the BM should not depend on frequency but cited evidence that the MOCR has a larger effect at high than at low frequencies. They therefore interpreted this finding as evidence against an explanation in terms of fast-acting compression central to the BM and in favour of the operation of the efferent system. However, in their study, both the auditory filter bandwidth and the number of masker components were greater at higher signal frequencies. As argued in the “Introduction” and elsewhere (e.g. Carlyon and Datta, 1997a; Summers, 2000), the peakiness of the auditory filter output and therefore the effects of any fast-acting compression will depend on the number of masker components interacting in the filter passband. Whilst acknowledging this point, Wojtczak and Oxenham (2009) noted that, although the phase effect for their off-frequency masker was not statistically significant for their 2-kHz signal, an off-frequency phase effect was observed by Oxenham and Ewert (2005) in simultaneous masking, using broadly similar stimuli. However, it is worth noting that phase effects are generally larger in simultaneous than in forward masking, presumably because the former paradigm allows the subject to “listen in the dips.” For example, with an 85-dB SPL on-frequency forward masker and a 2-kHz signal, the maximum phase effect observed by Wojtczak and Oxenham (2009) was about 12.5 dB, compared to 25 dB observed in simultaneous masking obtained with a broadly similar masker by Oxenham and Ewert (2005). It is also worth noting that Oxenham and Ewert’s (2005) off-frequency masker was low-pass filtered with a 6 dB/octave slope. This may have partly counteracted the effect of the lower slope of the auditory filter centred on the 2-kHz signal frequency, leading to more equal-amplitude components at the output of that filter. This in turn could have increased the peakiness in the 2-kHz auditory filter’s output.

We therefore repeated Wojtczak and Oxenham’s experiment with 2- and 6-kHz signal frequencies, but modifying the F0s to be a constant proportion of the signal frequency. These F0s were 66.7 Hz for the 2-kHz signal and 200 Hz for the 6-kHz signal. As shown in Table 2, the number of masker components and the ratio of the highest component to the signal frequency were the same for the two signals. Because auditory filter bandwidths are a roughly constant proportion of signal frequency, this would lead to approximately the same number of components interacting in the auditory filters centred on the two signal frequencies.

**TABLE 2:** Maskers used in experiment 2. The *left-hand column* shows the signal frequency in kHz followed by the masker F0 in Hz. The *next four columns* show the number of masker components, the lowest and highest masker component frequency (Hz) and the masker frequency range (Hz). The *final column* shows the range of values of *C* (Eq. 1) used

Fs (kHz)	F0	*N*	Low	High	Range	*C*
2	66.7	12	533.3	1333.3	800	−1 to +1
6	200	12	1600	4000	2400	−1 to +1

The masker level was again 85 dB SPL in each case. Because no forward masking data have been previously obtained with these combinations of masker F0, masker frequency range, and signals, we did not know in advance which values of *C* would lead to the most and least masking for each condition. The experiment was therefore performed with nine closely spaced values of *C* (−1, −0.75, −0.5, −0.25, 0, 0.25, 0.5, 0.75, and 1). Five normal-hearing listeners took part, three of whom had also participated in experiment 1. In all other respects, the methods were the same as in experiment 1.

### Results

**FIG. 2:**
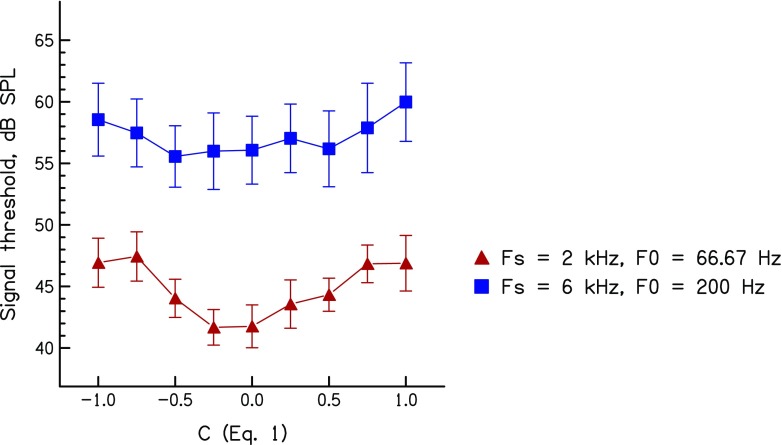
Forward masked thresholds as a function of *C*. Data obtained with a 2-kHz signal frequency and a masker F0 of 66.7 Hz are shown by *red triangles*. Results for a 6-kHz signal frequency and a 200-Hz masker F0 are shown by *blue squares*. *Error bars* show ±1 standard error of the group mean.

The results of experiment 2, averaged across the five listeners who took part, are plotted in Figure 2. It can be seen that, in both conditions, thresholds reach a minimum at a value of *C* equal to, or slightly below, zero. A two-way (*C* × signal frequency) repeated-measures ANOVA revealed main effects of masker phase (*F*(8,32) = 13.48, *p* < 0.003) and of signal frequency (*F*(1,4) = 7.75, *p* < 0.05), and there was a significant interaction that reflected the slightly different shape of the curves relating threshold to *C* at the two frequencies (*F*(8,32) = 4.25, *p* < 0.04). The effect of phase was significant when the data were analysed for each frequency separately (2 kHz: *F*(8,32) = 13.45, *p* < 0.002; 6 kHz: *F*(8.32) = 6.69, *p* < 0.019). Furthermore, the difference between the largest and smallest threshold across all values of *C*, calculated separately for each subject and signal frequency, was similar at 2 (6.9) and 6 kHz (5.5 dB) These differences did not differ significantly between the 2- and 6-kHz signals (*t*(4) = −0.9, *p* = 0.2). Clearly, masker phase has a substantial effect at both frequencies. The data show that, once the number of masker components has been equated between the two conditions, there is no indication that this effect is larger at 6 kHz than at 2 kHz. This finding is consistent either with some BM non-linearity (compression and/or suppression) affecting thresholds for off-frequency broadband maskers or compression at a more central stage. It is not consistent with the Wojtczak and Oxenham’s (2009) hypothesis that there is a frequency-dependent MOCR effect that results in the phase of off-frequency maskers influencing thresholds only at 6 kHz.

One difference between the maskers used for the two signal frequencies was that, for the 2-kHz signal, the masker F0 was three times lower (66.7 Hz) than that used for the 6-kHz signal. As noted in the “Method and Rationale” section above, this was done so as to roughly equate the number of components interacting in the auditory filter centred on each signal. It is possible that the longer periodicity associated with the lower-F0 masker had some additional effect on forward masked thresholds produced by the most-peaky masker, because, at the lower F0, neurons would have more time to recover between successive masker peaks. It is not clear though how this would modify the observed effect of phase curvature. Contemporary models of forward masking usually assume that the masker and signal are integrated using a sliding temporal window (Plack and Moore, 1990). If this is correct, then the greater recovery from refractoriness between masker peaks that occurs with the lower-F0 masker might increase its effectiveness. This would in turn reduce the observed difference between thresholds obtained with the most-peaky and (more effective) least-peaky masker. Alternatively, if forward masking resulted from long-term adaptation reducing the neural response to the probe, this adaptation might be smaller when neurons have a longer time to recover between successive peaks. This would decrease the effectiveness of the more-peaky masker and increase the observed phase effect. Regardless of which, if either, of these factors affects the forward masked thresholds observed here, it is important to remember that neither invoke the operation of the efferent system.

## **EXPERIMENT 3. OFF-FREQUENCY BACKWARD MASKING**

### Method and Rationale

Experiment 2 showed that the effect of phase on off-frequency masking was consistent either with a BM non-linearity or with more central fast-acting compression. As argued above, it is not consistent with an MOCR effect that is larger at 6 kHz than at 2 kHz. However, although Wojtczak and Oxenham (2009) cited physiological evidence for the frequency dependence of the MOCR, it is not always straightforward to generalise from animal to human experiments, and it is possible that, in humans, the MOCR does not differ markedly across this frequency range. It is also worth noting that the MOCR is just one of a class of possible mechanisms whereby masker phase exerts its influence via the response of neurons tuned to the masker, without changing the amount of masker excitation in neurons tuned to the signal frequency. One other such mechanism, MEM activation, was mentioned in the “Introduction” and investigated by Wojtczak et al. (2015). Another possibility could arise if forward masking were due to adaptation; when the BM response to the masker is very peaky, adaptation might recover during the low-amplitude portions between each peak. If this were true, then two maskers might produce the same total excitation, when averaged over the masker duration, but the “peakier” masker would produce less adaptation and therefore less forward masking.

Experiment 3 tested for an effect of masker phase on backward masking, which cannot be due to the MOCR, the MEM, adaptation, or indeed by any mechanism dependent on an after-effect of the masker. The method and stimuli were the same as in the off-frequency 6-kHz condition of experiment 1, except that the signal was presented immediately before the masker instead of immediately after it. Seven normal-hearing listeners (as determined by pure tone thresholds, described in experiment 1) took part, four of whom (S1–S4) had also participated in experiment 1. In order to calculate the across-subject correlation between the phase effects in forward and backward masking, we re-used those listeners’ forward masking data from the corresponding condition of experiment 1. For the three new listeners, we measured both forward- and backward-masked thresholds at each phase. In order to control for the possibility that the backward masker in one interval of a trial could influence the response to the probe in the next interval, we also measured forward-masked thresholds, using the same parameters as above, but with a masker-probe gap of 570 ms. This value was equal to the inter-trial interval in the backward masking experiment. Four subjects (S1, S3, S4, and S5) took part in this control experiment.

### Results

The results of experiment 3 are shown in Table 3, with the difference between thresholds obtained at *C* = −1 and *C* = 0 shown in bold in the third and sixth columns for backward and forward masking, respectively. It can be seen that this off-frequency phase effect varies substantially across listeners in both cases. The effect reached significance for forward masking and just failed to reach significance for backward masking, as can be seen by comparing the average size of the phase effect and the 95 % confidence intervals in Table 3. The size of the phase effect did not differ significantly between forward and backward masking (paired sample *t* test, df = 6, *p* = 0.18). Table 3 also shows that no listener demonstrated a substantial phase effect in the control experiment. This was true even for subjects S1 and S5, who both showed a large phase effect in backward masking. Indeed, for three of the four listeners who took part in the control experiment, masked thresholds were within 3 dB of those obtained in quiet (shown in the right-most column).

**TABLE 3:** Masked thresholds for the seven subjects of experiment 3 in backward and forward masking. Masked thresholds, as well as detection thresholds in quiet, are also shown for the four subjects who took part in the control forward masking experiment with a long masker-signal gap. Data for a given value of *C* (−1 or 0) are shown in *plain type*, with the threshold difference shown in *bold type*. The means and 95 % confidence intervals are shown in italics for the main experiment in the *bottom two rows*

	Backward			Forward			Fwd, long gap			Quiet
	−1	0	Diff	−1	0	Diff	−1	0	Diff	
S1	44.5	35.0	**9.5**	63.5	49.2	**14.3**	29.0	28.3	**0.7**	**24.3**
S2	57.9	46.4	**11.4**	57.9	50.0	**8.0**				
S3	38.9	37.3	**1.6**	56.2	47.6	**8.6**	30.8	32.4	**−1.6**	**28.0**
S4	47.4	54.7	**−7.3**	44.1	44.6	**−0.5**	35.6	36.7	**−1.1**	**34.3**
S5	72.3	47.8	**24.4**	69.7	50.5	**19.3**	37.0	36.7	**0.3**	**35.6**
S6	46.8	42.6	**4.2**	47.4	39.0	**8.4**				
S7	37.6	38.1	**−0.5**	52.9	47.5	**5.3**				
Mean	*49.3*	*43.1*	***6.2***	*56.0*	*46.9*	***9.1***				
95 % CI	*9.0*	*5.2*	***7.5***	*6.6*	*3.0*	***4.7***				

**FIG. 3:**
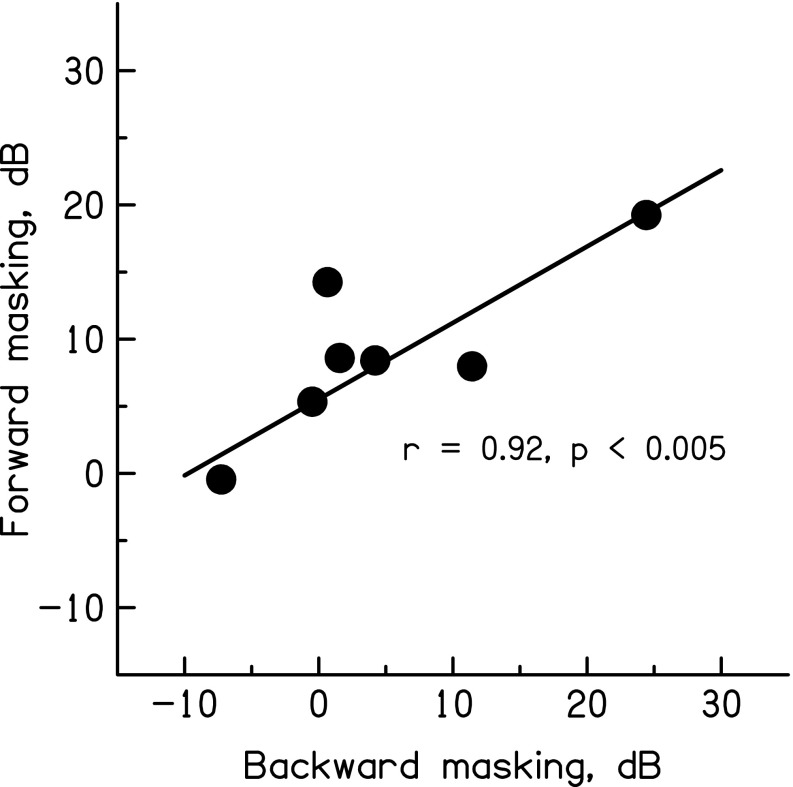
Across-subject correlation between the effect of masker phase curvature—defined as the difference in masked thresholds between masker phase curvatures (“*C*”) of −1 and 0 in off-frequency forward vs. backward masking. Each *circle* shows the data for one listener.

The reasons for the variability across listeners are unclear but might be due to differences in the extent to which subjects listened off-frequency. The masker components were all more than half an octave below the signal, and the optimal filter to detect the signal will depend on the slope of the listener’s audiogram above 6 kHz, the bandwidths of his/her auditory filters above 6 kHz, and the steepness of the upper slope of the masker’s excitation pattern. This latter factor will, for the stimuli used here, depend both on the magnitude and the phase responses of the auditory filters tuned to frequencies above the masker cut-off. Any or all of these factors could differ across listeners. What is clear, though, is that those listeners who showed a substantial phase effect in forward masking also showed a large effect in backward masking. This is illustrated by the scatter plot in Figure 3, which shows the difference between thresholds obtained at *C* = −1 and *C* = 0 for each listener in forward masking on the ordinate and in backward masking on the abscissa; it can be seen that the phase effects in forward and backward masking correlate significantly (Pearson’s *r* = 0.92, df = 5, *p* < 0.005). This correlation remained significant when one listener, who showed no phase effect in forward masking and a negative phase effect in backward masking, was removed (*r* = 0.88, df = 4, *p* < 0.05).

## **DISCUSSION**

### Mechanisms Underlying Phase Effects

When the frequency range of the masker encompasses that of the signal (on-frequency masking), the size of the masker phase effect for harmonic tone maskers is greatest at high masker levels (Carlyon and Datta, 1997a; Gockel et al., 2003; Moore et al., 2004; Stainsby and Moore, 2007), low F0 s (Gockel et al., 2003) and, for a given F0, higher signal frequencies (Moore et al., 2004; Wojtczak and Oxenham, 2009; present study). All of these findings are consistent with the fact that the maximum difference in crest factor at the output of a filter depends on the number of components interacting within that filter. In addition, as Carlyon and Datta (1997a) pointed out, the increased phase effect at high masker levels is consistent with, but is not necessarily entirely due to, the operation of fast-acting BM compression. Carlyon and Datta’s (1997a) measurements were obtained with a signal frequency of 1000 Hz and a masker F0 of 100 Hz. This led to a maximum difference in the levels of *S*+ vs. *S*− maskers, necessary to mask the same signal, of between 10–15 dB for most of their listeners. To determine whether this effect was consistent with peripheral compression, they modelled the output of an auditory filter centred on 1000 Hz for two 100-Hz F0 complex tones, designed to produce outputs that differed maximally in peakiness given the limited bandwidth of the filter. They found that even a power law compression with an exponent of 0.1 predicted only a 10-dB difference, which was at the lower end of the differences observed experimentally. Subsequently, Gockel et al. (2003) observed a difference of up to 35 dB in the effective levels of a cosine-phase and a random-phase complex with a 62.5 Hz F0. Gockel et al. argued that their effect was too large to be due to BM compression alone and suggested that suppression might also play a role. Specifically, when a stimulus produces a peaky output across a wide range of auditory filters, those outputs might be reduced by BM suppression. It is worth noting that suppression and compression can be viewed as manifestations of the same BM non-linearity, and that, as the masker level increases, the filter bandwidths become broader and their output becomes even peakier, the effects of “compression” will also increase. This will in turn be reflected in a reduction in the slope of the BM input-output curve, and hence can be viewed as a form of compression, albeit one that is more severe than that observed with sinusoidal stimuli. Hence, it is perhaps more accurate to state that phase effects in forward masking, observed with broadband stimuli, can be too large to be accounted for by compression *as measured and observed for sinusoidal stimuli*. As noted in the “Introduction,” for succinctness, we use the term BM non-linearity throughout most of this article.

Evidence for a major role for BM non-linearity in the phase effect observed for on-frequency forward masking comes from the finding that those effects are reduced or absent in listeners having a moderate sensory hearing loss (Moore et al., 2004; Stainsby and Moore, 2007). This finding could in principle be due to the phase curvature of auditory filters being affected by sensory hearing loss. Oxenham and Dau (2004) provided some evidence that this is not the case. They measured simultaneous-masked thresholds for a sinusoidal signal as a function of *C* for a signal frequency of 250 Hz and a 12.5-Hz F0, and for a signal frequency of 100 Hz and F0s of both 12.5 and 100 Hz. For the 12.5-Hz F0 the minimum threshold occurred at approximately the same value of *C* for normal-hearing and hearing-impaired listeners, at both signal frequencies. Hence, the absence or reduction of phase effects in forward masking, observed for hearing-impaired listeners, provides some evidence that such effects do indeed reflect a BM non-linearity. However, one *caveat* is that, for the condition most similar to that studied here—a 100-Hz-F0 and a 1-kHz signal frequency—the threshold vs. phase function was too shallow to reveal a clear minimum for most hearing-impaired listeners. Furthermore, Shen and Lentz (2009) have argued that the effect of phase curvature on simultaneous-masked thresholds can be modelled using an auditory filter whose phase curvature is not constant across its passband but instead tends to zero for frequencies far below its CF. If this were the case then, due to the different filter magnitude responses in normal and impaired ears, one might expect different effects of masker phase curvature for impaired and normal-hearing listeners.

It is also worth noting evidence that some listeners with quite substantial hearing loss do show differences in forward masking between random-phase and cosine-phase tones (Moore et al., 2004; Stainsby and Moore, 2007). It may be that non-linearities either in the transduction process or in neural responses are not sufficient to produce forward masking phase effects for normal-hearing listeners but can do so in hearing-impaired listeners whose broader auditory filters permit the interaction of a greater number of components. It may also be that, even in normal-hearing listeners, the effects of neural/transduction compression could contribute to phase effects for maskers with very low F0s and/or at high signal frequencies.

Another possibility is that transduction/neural compression contributes to the phase effects observed with off-frequency maskers in the present study and that of Wojtczak and Oxenham (2009). Note that Wojtczak and Oxenham rejected this observation because they observed off-frequency phase effects only for the 6-kHz signal frequency, but the results of experiment 2 shows that the effect is at least as large for a 2-kHz signal frequency once one equates the number and their spacing as a proportion of signal frequency. However, one should not rule out a possible role for the BM non-linearity, given that its effects are likely to be more marked for peaky harmonic complexes than for the pure tones used in most psychophysical and biomechanical experiments that investigate it. It is also worth noting that upper edges of our off-frequency maskers were only 0.58 octaves below the signal frequency, which is not much more than the 0.5 octaves over which the BM response is non-linear. Recio and Rhode (2000) observed that this range was somewhat larger at a characteristic frequency of 5.5 kHz than at 14.5 kHz, and so it is also possible that the extent of this non-linear region depends on frequency. Certainly, the most parsimonious explanation for the effect of masker phase on forward masking is that it primarily or exclusively reflects the operation of the cochlear non-linearity, and that these effects are larger than and apply to a wider range of signal frequencies than would be estimated solely from experiments that employ sinusoidal stimuli.

## **SUMMARY**

(i) Masker phase has a strong effect on forward masked thresholds, both for on- and off-frequency maskers. When the F0 is 100 Hz, as in the present study, the effect is larger at a 6-kHz than at a 1-kHz signal frequency, especially for a 200-ms (compared to a 30-ms) masker. The larger phase effect at the higher signal frequency is consistent with the greater number of components interacting within an auditory filter centred on the signal.
(ii) The effect of masker phase on off-frequency forward masking is roughly similar for 2- and 6-kHz signals, provided that one equates the number of masker components and their frequency spacing relative to the signal frequency.
(iii) The phase effect in off-frequency masking correlates strongly across subjects in forward and backward masking. This provides strong evidence against an explanation in terms of the MOCR or the MEM, or any other explanation that depends on an aftereffect of the masker.
(iv) The results presented here and elsewhere are consistent with a primary role for the BM non-linearity, with a possible smaller contribution by fast-acting compression during transduction or neural processing
